# An Anomalous Case of Bubonocele, with Its Somewhat Novel Sequelæ

**Published:** 1857-03

**Authors:** H. P. Strong

**Affiliations:** Beloit, Wis.


ART. II.—*An Anomalous Case of Bubonocele, with its somewhat novel Sequelæ.* By H. P. STRONG, M. D., Beloit, Wis.

Allow your correspondent his prerogative of omitting the dates, name, age, temperament, habits, vocation, &c., which are usually given in routine writing, and permit him to proceed directly to the case under consideration.

I will say this much, that a lady of about middle age, had the audacity to send a messenger for me, not *more* than ten years since, to take charge of her case, which was, as the messenger expressed it, "in a bad condition."

In giving a description I shall be obliged to rely partially on the statements of herself and friends, all of which were corroborated by the condition of things as found by myself at my first visit.

For several years she had been afflicted with a small hernia, which subjected her to no more than the ordinary inconvenience attending rupture, until about three weeks previous to her coming under my care. At, or about this time, she thoughtlessly lifted quite a heavy weight, and soon felt uneasiness and pain in the abdomen, and after an attempt at reduction of the hernia in the evening, found that she "could n't get it back." There were no very urgent symptoms demanding attention, and surgical aid was not solicited until the next day, when their family physician was called. It was diagnosed as a case of femoral hernia—probably reducible by taxis. The symptoms were mild, and cathartics, with taxis, were *faithfully* tried until the *sixth day of strangulation*, without any visible effect, save that which we would naturally expect upon the patient's vitality. Counsel was called, and an operation was advised, accepted, and performed for femoral hernia.

The tumor was small and soon returned, (as was supposed by the surgeon,) into the cavity of the abdomen. Some time after the operation (how long I do not know) she had one small dejection, which was rather small in quantity. The inflammatory action following the operation, was comparatively slight, yet the incision did not show a disposition to heal. The general symptoms were neither very favorable nor unfavorable.

In a few days (perhaps five or six) after she was cut, there appeared on the skin, a bluish-red prominence, situated from two to three inches above and to the outside of the incision. This was noticed by the attendant surgeon for a time, and then opened with a bistoury. There was but little discharge. On inspection of the opening thus made, there was found, as the lady said, an ash-colored membranous slough, which was seized by the forceps and extracted, that appeared to be "a portion of the bowel three or four inches in length." Immediately following this was a discharge of thin pus, serum, and liquid fæces, by both the former and the latter opening, gradually increasing up to the time when I first saw and took the case under my care. For convenience I will designate the latter as the fistulous opening, and the former as the incision.

I found the patient much emaciated, with but little appetite and considerable thirst. The pulse was weak, and varying from 110 to 120, presenting the feel peculiar to atonic peritoneal inflammation. She was vomiting at short intervals, a darkish colored fluid, in small quantities. Her countenance was rather pinched and cadaveric in its expression. The right side of the abdomen extending down on to the thigh a short distance, was red, hot, quite hard and painful, plainly showing that all the tissues, the peritoneum, muscles, fascia, cellular tissue and skin, were involved in the inflammatory action. The inside of the thigh, the peritoneum and right side of the vulva, were considerably excoriated by the constant discharge. She complained a great deal of the pain and scalding and could bear but little pressure on the iliac or inguinal regions. The fistulous opening was now one inch in length, and communicated directly with the cavity of the abdomen by way of the *direct inguinal* canal. Out of this opening there hung an ash-colored membranous looking substance, about one-half an inch in length, and I noticed that the fæces were discharged *through* this as through a tube. In a few days this slough came away, and was about six inches in length. Slight pressure above the incision would cause the fæces to gurgle out.

There were no external applications in use when I first visited the patient, and from want of information I cannot give the constitutional treatment pursued.

Of course, the prognosis by myself and others who saw the case, was *very* doubtful, yet knowing somewhat the inherent vitality and recuperative modeling power of nature, I thought an effort to save life warranted by a shaddow of success, even if there was left as a result, that which choice would dictate as akin to death,—an artificial anus.

I ordered Dover's powder, quinia, and brandy, as anodyne and stimulants, and for external application a hop poultice applied often and warm.

Under this treatment there was a manifest improvement from the first, both in the general condition and the local inflammation. For two weeks there was a general improvement and a gradual diminution in the quantity of pus and serum discharged. After this, nature seemed to make an effort at restoration of the parts to their normal condition. The symptoms of internal local inflammation increased for a few days, and there followed a large discharge of pus by both the fistulous opening and incision. The quantity discharged was from eight to ten ounces a day, for several days. Simultaneously with this, the discharge of fæces diminished until they ceased altogether, and she had a passage *per anum*, attended with considerable pain. The amount of pus and serum discharged per day, was about the same for seven or eight days, and then it began to diminish in quantity and consistence. After a few weeks the discharge had changed from pus to a straw-colored serum, free from pus globules, and much less in amount.

After the natural passage was first established, the incision began to granulate and heal, until it was so far closed that nothing but a very small fistula was left. The fistulous opening, after a time, followed the same course, and when I last saw the patient they were neither of them entirely closed, and both discharged serum invariably, but at no time very large quantities.

When the natural effort at restoration had taken place, and the discharge of pus began to diminish, her appetite and strength rapidly improved. In about three months after her confinement to the bed, she was dressed, and walked about the room a little, but I believe she has not yet been able to attend to her household duties. Exercise creates pain and soreness in and about the abdomen on the right side, which is the result of the long-continued inflammatory action in the muscles of those parts. She has a motion of her bowels during every forty-eight hours, and always attended with pain, referred to the vicinity of the former difficulty, and with quite a rumbling noise, which, upon auscultation, appears to be limited to a straight line drawn from one fistula to the other.

*Remarks.* When I first saw this patient and obtained a description of her case, I was strongly impressed with the belief that two, and perhaps three, errors had been committed in its management, as, perhaps, others may think, who take the case as presented.

The first was, that an almost unprecedented time was allowed to intervene between strangulation and the operation,—a time unwarranted by all the experience and writings of the most reliable authorities.

Mr. Hey, whose authority to speak is undoubted, says: "I can scarcely speak in too strong terms of the necessity of an early recourse to the operation, as the most effectual method of preserving life in this dangerous disease. If Mr. Potts' opinion be true, that the operation, when performed in a proper manner, and in due time, does not prove the cause of death oftener than, perhaps, once in fifty times, it would, undoubtedly, preserve the lives of many, to perform it almost as soon as the disease commenced, without increasing the danger by spending much time in the use of means which cannot be depended upon for a cure. I have since seen the disease prove fatal in about twenty-four hours.

"I have now, at the time of writing this, performed the operation thirty-five times, and have often had occasion to lament that I had performed it too late, but never that I had performed it too soon."

Experience confirms the truth of the following passage of Mr. Potts' Treatise: "A hernia with painful stricture and stopping of stools, is one of those cases in which we can seldom stand still even for a short space of time; if we do not get forward we generally go backward; and whatever does no good, if it be at all depended upon, certainly does harm, by occasioning an irretrievable loss of time."

Samuel Cooper remarks, that it is generally better to operate too early, than too late, and that the necessity of having a speedy recourse to the operation, as soon as the surgeon has unsuccessfully put into practice some of the most efficacious and least dilatory plans of treatment, is indicated by daily experience. This is more particularly the case, since we find it recorded by almost every writer upon the subject, that the intestine is often found in a state of mortification a very few hours after its first protrusion from the cavity of the abdomen.

Pirrie gives the rule that if the patient be brought well under the influence of chloroform, and if on the decided, skillful, and thorough employment of the taxis, the hernia cannot be returned, the surgeon may reasonably conclude that the constriction is too great to be overcome by any means short of an operation. He should, therefore, spare the patient the danger resulting from delay and unnecessary handling, and at once proceed to the operation.

Twenty-four hours is the time fixed by Sir A. Cooper, S. Cooper, Bransby Cooper, Larrey, Pott, Wilmer, and Sir C. Bell, as the extent we can safely go in *any* case before having recourse to the operation, and in many cases occurring in the young and vigorous, even that time will ensure a fatal termination.

I have given these authorities hoping thereby to impress *some* of the profession who I fear do not now feel it, with a sense of their responsibility and the necessity for timely action.

This operation, with ovariotomy, tracheotomy, and laryngotomy, and some others, have discredit cast upon them, I think, and thereby their efficiency impaired by being resorted to at too late an hour.

The second point I would notice is the error in diagnosis.

It was diagnosed as a case of simple femoral hernia, yet from the description and appearance at the time I first saw it, and the development from that time onward, I was perfectly convinced that it was not a *simple*, but a *complicated* case. What those complications were can only be demonstrated from suppositions founded on the changes and transformations which had taken place during the three weeks prior to my acquaintance with the case.

The irregularity was somewhat analogous to that of three cases reported by J. F. South, in his notes to Chelius's Surgery, vol. ii., p. 338. My belief in this case is, that there was a double stricture, but I shall not attempt to decide whether it was from the original sac's bursting and forming two with a kind of hour glass contraction, or from a second stricture in the parts through which the sac passed. I incline to the latter opinion, however. Originally, I think it must have been a case of *indirect* inguinal hernia; but at the time of strangulation it had become *direct*, from its long standing. The fistulous opening that formed, communicated with the cavity of the abdomen by the direct canal, and here the original stricture must have been, which was not relieved by the operation; hence, the ulceration and sloughing—nature accomplishing what art should have done. No doubt the bowel passed out of its usual course and did not appear as a tumor until near the femoral ring where there was a second stricture. The operation relieved this, and instead of the bowel being returned into the abdomen, it was only pushed back toward the inguinal ring. The fact that both the fistulous opening and the incision discharged serum, pus, and fæces, argues strongly that there was a communication between them, and sustains my opinion.

It is true the patient had a stool after the operation, but to my mind this does not materially affect the supposition; for it is equally true that the stool was small and the only one she had previous to the ulceration. That the upper portion of the illeum was strangulated is proved, I think, by the fact that food and drink taken could be detected in one and a-half hours, at the fistula, and sometimes less. This stool may have been the secretions formed below the strangulated portion.

The third probable error was the neglect, on the part of the surgeon, to find out the condition of the bowel, and, if necessary, form an artificial anus, by stitching the bowel to the parts around the incision. This is the rule to be followed in all cases; but the result in the case under consideration, proved that the neglect was beneficial rather than hurtful.

The question naturally occurs to the mind—what were the sloughs spoken of in the description of the case? Perhaps I shall find more difficulty in convincing my incredulous brethren of the justice of my conclusions in this, than in anything else connected with the case. I am bound in honesty to acknowledge my credulity in the wisdom and power of nature in accomplishing results equal to the emergency; yet the way in which this was done I shall leave for speculation.

I answer, then, according to the oath, "to the best of my knowledge and belief," the sloughs were nothing more nor less than *portions of the bowel*, amounting in all, from six to ten inches. Of that which was extracted by the surgeon, with the forceps, I cannot speak so positively, but my belief is, that it was a portion contained in the sac lying in the canal formed between the fistulous opening and the incision. Of that spoken of as appearing at the mouth of the fistulous opening I am more confident. But most will say, "you mistake, it was *omentum!*" Others, that it was cellular tissue, peritoneum, or something else—anything but the bowel. In answer to all these, I will say that I examined it closely and found it tubular and regular in form—the size of the intestine, and so far as I could judge, of the same structure. Furthermore, the fæces, as I before stated, were discharged *through* this tube and not *around* it, which was not true of the discharge of pus and serum, they being discharged *around*, and not *through* the tube. This slough came away with the dressing, and was destroyed in my absence, else it would have been preserved for further examination.

From the description of her sensations while lying on her back or when she turns on either side, I judge that the two ends of the intestine are united to the inner walls of the abdomen, at points nearly corresponding to the openings. May there not be an artificial passage formed between the two ends, or did they come together and unite?

The two fistulas are still open, and probably always will be. Should they close, she would have ascites as a consequence, without doubt.

If the opportunity for a *post-mortem* should ever occur, I will investigate the case and make known the condition of the parts; meanwhile, if my deductions are wrong, I would ask my brethren to be as charitably disposed as Sancho Panza was when governor of his island, and adopt his rule, that "jusought always lean to the side of mercy."

